# Cost-Effectiveness Analysis of Atezolizumab Versus Chemotherapy as First-Line Treatment for Metastatic Non-Small-Cell Lung Cancer With Different PD-L1 Expression Status

**DOI:** 10.3389/fonc.2021.669195

**Published:** 2021-04-27

**Authors:** Guoqiang Liu, Shuo Kang, Xinchen Wang, Fangjian Shang

**Affiliations:** ^1^ Department of Pharmacy, The Third Hospital of Hebei Medical University, Shijiazhuang, China; ^2^ Laboratory of Pathology, Hebei Cancer Institute, The Fourth Hospital of Hebei Medical University, Shijiazhuang, China; ^3^ Department of General Surgery, Hebei Key Laboratory of Colorectal Cancer Precision Diagnosis and Treatment, The First Hospital of Hebei Medical University, Shijiazhuang, China

**Keywords:** atezolizumab, non-small-cell lung cancer, cost-effectiveness, chemotherapy, first-line treatment

## Abstract

**Background:**

Atezolizumab could significantly improve clinical outcomes and was associated with less toxicity compared with chemotherapy as the first-line treatment of PD-L1-selected patients with EGFR and ALK wild-type metastatic non-small-cell lung cancer (NSCLC). However, the economic outcomes remain unclear yet in China. This study aimed to investigate the cost-effectiveness of atezolizumab versus chemotherapy as first-line therapy for metastatic NSCLC with different PD-L1 expression status from the Chinese health sector perspective.

**Methods:**

A decision-analytic model was conducted to evaluate the economic outcomes for the first-line treatment of EGFR and ALK wild-type metastatic NSCLC with atezolizumab and chemotherapy in high PD-L1 expression, high or intermediate PD-L1 expression and any PD-L1 expression populations, respectively. The efficacy and safety data were obtained from the IMpower110 trial. Cost and utility values were gathered from the local charges and published literatures. Incremental cost-effectiveness ratio (ICER) was estimated. A scenario analysis for a patient assistance program (PAP) was conducted. One-way and probabilistic sensitivity analyses were performed to explore the robustness of the model results.

**Results:**

Atezolizumab yielded additional 0.91 QALYs, 0.57 QALYs, 0.42 QALYs in comparison with chemotherapy, and the ICERs were $123,778.60/QALY, $142,827.19/QALY, $168,902.66/QALY in the high PD-L1 expression, high or intermediate PD-L1 expression, and any PD-L1 expression populations, respectively. When PAP was available, the ICERs were $52,414.63/QALY, $52,329.73/QALY, $61,189.66/QALY in the three categories of PD-L1 expression status populations, respectively. The ICERs were exceed the willingness-to-pay (WTP) threshold of $30,828/QALY (three times of per capita gross domestic product of China in 2019) in China. One-way sensitivity analyses suggested that the cost of atezolizumab played a vital role in the model outcomes, and the probabilistic sensitivity analyses showed atezolizumab was unlikely to be cost-effective at the WTP threshold regardless of PD-L1 expression status and whether the PAP was available or not.

**Conclusions:**

Atezolizumab as first-line treatment for PD-L1-selected metastatic NSCLC patients without EGFR mutations or ALK translocations is unlikely to be cost-effective compared with chemotherapy regardless of PD-L1 expression status in the Chinese context.

## Introduction

The Global Burden of Disease Study reported that lung cancer is one of the leading causes of non-communicable disease burden around the world (1). The incidence and mortality of lung cancer were ranked first among all cancers in China (2, 3). In 2015, the costs of treating lung cancer in China accounted for 0.6% of the total health expenditure (4). Approximately 85% of lung cancer is non-small-cell lung cancer (NSCLC). Because early disease is typically asymptomatic, up to 61% of patients have progressed to advanced stage at the time of diagnosis, which has inferior prognosis with a five-year survival rate of 18% (5, 6). Platinum-based chemotherapy has been recommended as the standard of care for the first-line treatment of metastatic NSCLC patients with epidermal growth factor receptor (EGFR) and anaplastic lymphoma kinase (ALK) wild-type in China (7). Recently, the introduction of immune checkpoint inhibitors (ICIs) demonstrated the reactivation of the antitumor functions of T cells through inhibiting the programmed cell death-1 (PD-1) and programmed cell death receptor ligand-1 (PD-L1) pathway through inhibiting the programmed cell death-1 (PD-1) and programmed cell death receptor ligand-1 (PD-L1) pathway (8–10). And immunotherapy have changed the treatment paradigm of patients with metastatic NSCLS due to its preferable clinical efficiency and safety profile (11–17).

Atezolizumab is an anti-PD-L1 monoclonal antibody. In comparison with chemotherapy, the IMpower110, an open-label, randomized phase 3 trial, demonstrated atezolizumab was less toxicity and significantly reduced the risk of disease progression or death by 37% (hazard ratio (HR), 0.63, 95% CI: 0.45-0.88), 33% (HR, 0.67, 95% CI: 0.52-0.88), and 23% (HR, 0.77, 95% CI: 0.63-0.94) for EGFR and ALK wild-type metastatic NSCLC patients who had not previously received chemotherapy with high PD-L1 expression, high or intermediate PD-L1 expression, and any PD-L1 expression, respectively (18).

Although atezolizumab brings clinical benefits to metastatic NSCLC, high price of atezolizumab urges us to pay more attention to the necessity for cost-effectiveness analysis in order to clear whether its high cost reflects its potential benefits in terms of value especially for resource-limited countries such as China (19–21). The objective of our analysis was to investigate the cost-effectiveness of atezolizumab versus chemotherapy as first-line therapy for EGFR and ALK wild-type metastatic NSCLC with different PD-L1 expression status from the Chinese health sector perspective.

## Materials and Methods

### Analytical Overview and Model Structure

**Figure 1:**
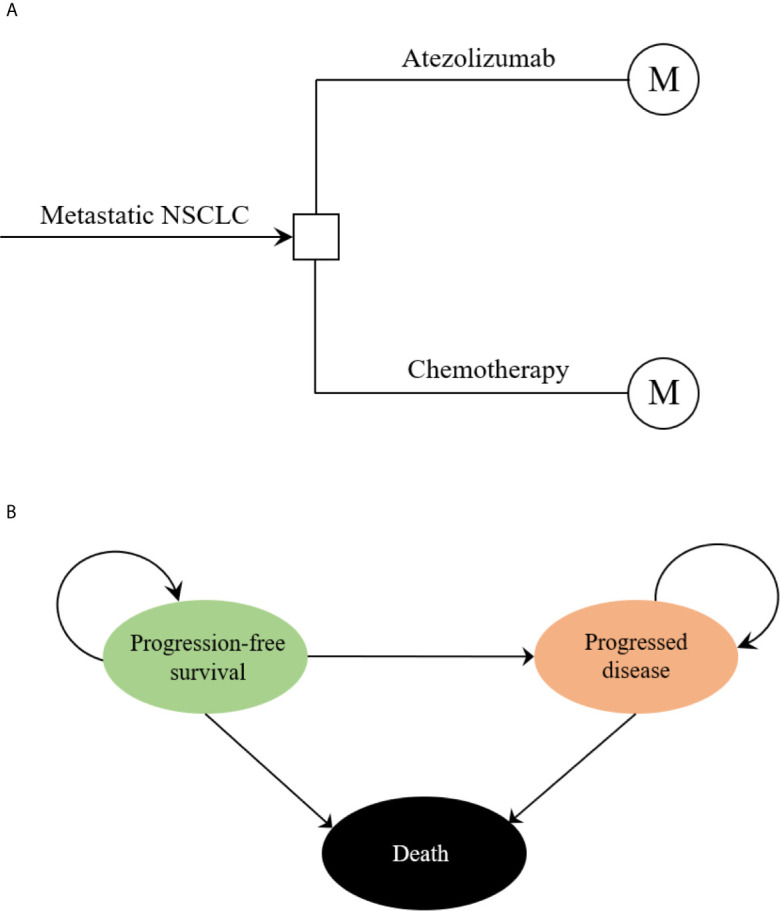
The structure of the **(A)** decision tree and **(B)** Markov model. NSCLC, non-small-cell lung cancer.

The hypothetical target population cohort was consistent with the patient characteristics of the IMpower110 trial, PD-L1 expression positive patients who had metastatic non-small-cell lung cancer (NSCLC) with EGFR and ALK wild-type and did not previously receive chemotherapy were eligible (18). A combined mathematical model was developed to assess the economic outcomes of the potential competing first-line treatment strategies. The decision tree model demonstrated a clear process of the decision-making. (**Figure 1A**). In the hypothetical target population cohort, a Markov process was conducted to project disease course of metastatic NSCLC, which included three mutually exclusive health states: progression-free survival (PFS), progressed disease (PD), and death (**Figure 1B**). The initial health state for all patients was PFS, the Markov cycle length was 3 weeks in keeping with the treatment schedule reported by IMpower110 trial, and the time horizon for the model was 10 years. During each Markov cycle, patients would either remain in their assigned health state or redistribute to a new health state according to time-dependency transition probabilities which were based on the results of the IMpower110 trial.

These following outcomes included costs, life-years (LYs) and quality-adjusted life-years (QALYs) were estimated. Costs are reported in 2019 US dollars (US $1= CNY ¥6.899), costs and QALYs were discounted at an annual rate of 5%, according to the Chinese guidelines for pharmacoeconomic evaluations (22). We estimated the incremental cost-effectiveness ratio (ICER), presented as cost per additional QALY gained, to judge cost-effective of the two treatments. Three times of the per capita gross domestic product (GDP) of China in 2019 (US $30,828/QALY) was used as the willingness-to-pay (WTP) threshold, in line with Chinese guidelines for pharmacoeconomic evaluations and WHO recommendations (23). The mathematical model was implemented using Microsoft Excel 2019 software (Microsoft Corporation, Redmond, WA), and statistical analyses were conducted using R software (version 3.6.1, http://www.r-project.org).

### Clinical Data

**Figure 2:**
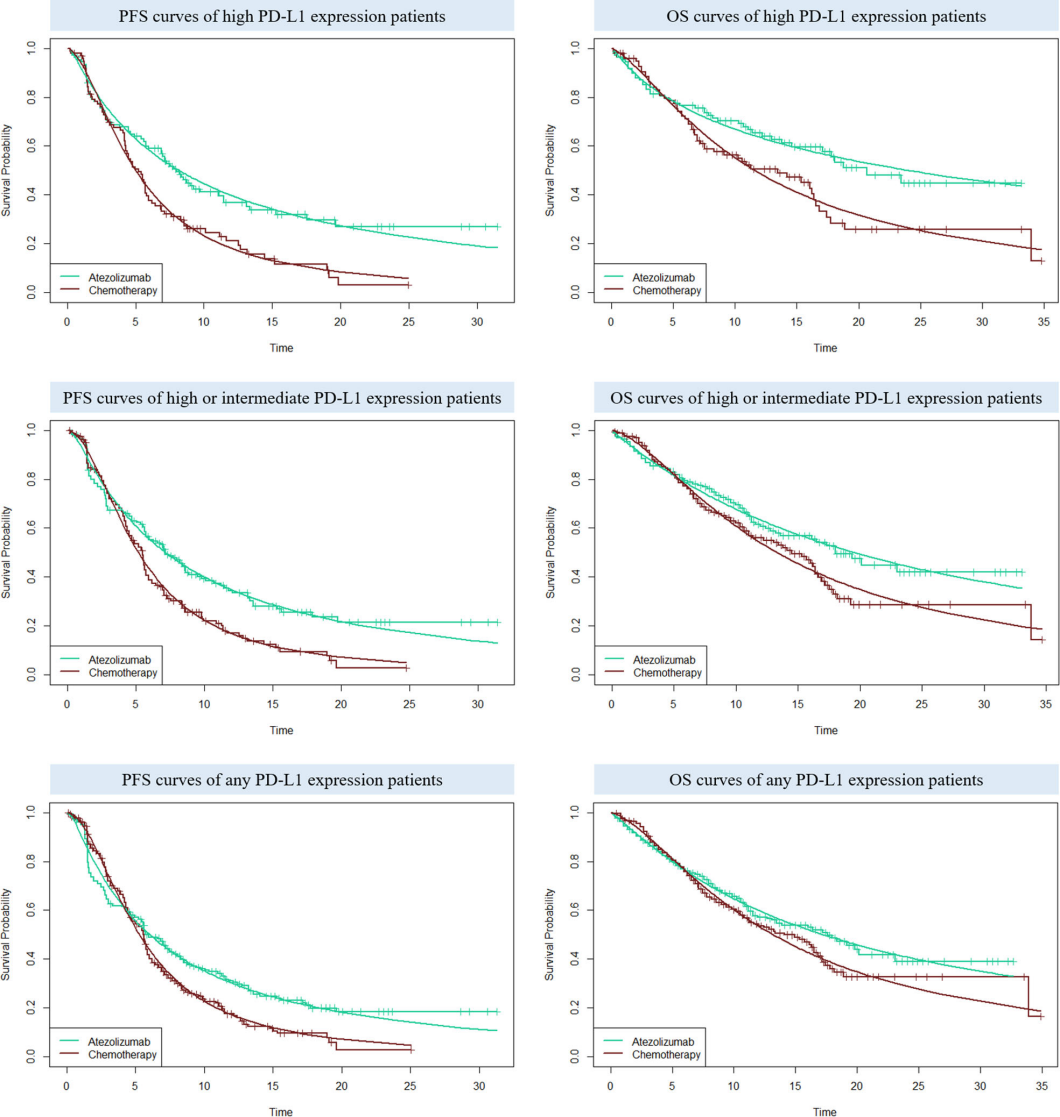
The comparison between the adopted fitting curves and the Kaplan-Meier (KM) curves in IMpower110 trial. PFS, progression-free survival; OS, overall survival; PD-L1, programmed death ligand 1.

For the following three categories of PD-L1 expression status populations: high PD-L1 expression, high or intermediate PD-L1 expression, and any PD-L1 expression, the clinical efficacy and safety data were obtained from the IMpower110 trial. PFS and OS curves were extrapolated over the model time horizon by using the standard statistical analyses developed by Guyot et al. (24). The GetData Graph Digitizer software (version 2.26; http://www.getdata-graphdigitizer.com/index.php) was used to gather the data points from the PFS and OS curves, then these data points were used to fit the parametric survival functions among: exponential, gamma, Weibull, log-logistic, log-normal, and gompertz. Goodness of fit was based on the visual inspection and Akaike information criterion (AIC), AIC values for the three categories of PD-L1 expression status populations were listed in **Supplementary Table 1**. The adopted model and estimated survival parameters related to PFS and OS curves for atezolizumab and chemotherapy were shown in **Table 1**. The comparison between the adopted fitting curves and the Kaplan-Meier (KM) curves in IMpower110 trial were shown in **Figure 2**. After the disease progressed, the data of patients who received second-line treatment was gathered from the IMpower110 trial.

**Table 1:** Survival model parameters fitting to the PFS and OS data from IMpower110 trial.

Populations	PFS		OS	
	Model*	Parameter	Model*	Parameter
High PD-L1 expression populations				
Atezolizumab	Log-normal	Meanlog=2.098; sdlog=1.494	Log-normal	Meanlog=3.180; sdlog=2.002
Chemotherapy	Log-logistic	Shape=1.724; scale=4.995	Log-logistic	Shape=1.407 scale=11.610
High or intermediate PD-L1 expression populations				
Atezolizumab	Log-normal	Meanlog=1.971; sdlog=1.312	Gompertz	Shape=-0.020; rate=0.043
Chemotherapy	Log-logistic	Shape=1.902; scale=5.188	Log-logistic	Shape=1.538; scale=13.316
Any PD-L1 expression populations				
Atezolizumab	Log-normal	Meanlog=1.798; sdlog=1.321	Log-logistic	Shape=1.117; scale=17.245
Chemotherapy	Log-logistic	Shape=1.919; scale=5.279	Log-logistic	Shape=1.503; scale=13.17

*adopted parametric survival function in the model; PFS, progression-free survival; OS, overall survival; PD-L1, programmed death ligand 1.

### Transition Probabilities

We used the obtained survival parameters and survival function of each PFS and OS curves reported in previous section to calculate the time-dependency transition probabilities of the Markov process. We assumed the transition probability of PFS to Death (P*_PFS to Death_*) was equal to the natural mortality, and the transition probability of PFS to PFS (P*_PFS to PFS_*) =1-S(t)/S(t-μ), where μ is the cycle length of Markov process, so the transition probability of PFS to PD (P*_PFS to PD_*) was 1-P*_PFS to Death_*-P*_PFS to PFS_*. Similarly, the transition probability of survival (including the patients in PFS and PD) to survival (P*_S to S_*) could calculate, then we could obtain the transition probability of PD to PD (P*_PD to PD_*) based on the following formulation:

$$\left[\left(n_{PFS}+n_{PD}\right)*P_{S\;to\;S}-n_{PFS}*P_{PFS\;to\;PFS}-n_{PFS}*P_{PFS\;to\;PD}\right]/n_{PD}$$

Where n*_PFS_*and n_*PD*_ indicate the number of patients in the PFS and PD in the last Markov cycle, respectively (25). The transition probability of PD to Death (P*_PD to Death_*) = 1-P*_PD to PD_*.

### Cost and Utility Values

Only direct medical costs were calculated in our model, including drug acquisition costs, costs of supportive care and routine follow-op, costs for the management of the treatment-related serious adverse events (SAEs, grade≥3), and costs of end-of-life care.

Based on the IMpower110 trial, patients would receive atezolizumab at a dose of 1200mg or platinum-based chemotherapy once every 3 weeks. In the chemotherapy group, patients with nonsquamous NSCLC received either cisplatin 75mg/m^2^ or carboplatin at an area under the curve 6mg/ml per min plus pemetrexed 500mg/m^2^, patients with squamous NSCLC received either cisplatin 75mg/m^2^ plus gemcitabine 1250mg/m^2^ or carboplatin at an area under the curve 5mg/ml per min plus gemcitabine 1000mg/m^2^ (18). We assumed a typical patient who had a body surface area (BSA) of 1.72m^2^ (height: 1.64m, weight: 65kg) to calculate the dose of chemotherapy (26). We only considered the costs of SAEs which with a difference≥3% between the two competing strategies in order to simplify the model, all costs related to SAEs were assumed to incur in the first cycle, and we tested the incidence rates and costs of SAEs in the sensitivity analyses. Atezolizumab patient assistance program (PAP) was conducted to improve the drug affordability. The atezolizumab PAP supports patients to pay for the first two cycles, then they would receive free atezolizumab for three cycles, and continue cycling. Although the PAP was currently only performed for patients with either extensive-stage small-cell lung cancer or unresectable hepatocellular carcinoma in China, we considered it for a scenario analysis in our study to explore the economic impact of this possible context in the future.

The health state utility values used in our model were gathered from the previously published study, the utility value of PFS and PD were 0.804 and 0.321, respectively (27, 28). The utility value of death was zero, and the disutility caused by SAEs were also computed in the model. These key model parameters were performed in **Supplementary Table 2**.

### Sensitivity Analyses

One-way sensitivity analyses and probabilistic sensitivity analyses (PSA) were used to test the robustness of the model outputs. In one-way sensitivity analyses, each relevant parameter was changed one-by-one to its preset lower and upper values to examine which parameter has substantial influence on the model outputs, the estimated range of each parameter was either based on the reported or estimated 95% confidence intervals in the previously studies or determined by assuming a range of ±25% of the base-case values if the data were not available. The ranges were shown in **Table 1**. The results of the one-way sensitivity analyses are displayed in the Tornado diagram. In the PSA, a Monte Carlo simulation of 1,000 iterations was conducted by simultaneously sampling the model parameters from their pre-specified statistical distributions. Gamma distribution was used for costs, log-normal distribution was used for hazard ratios, and beta distribution was used for incidence rates, proportions, and utility values, according to the ISPOR-SMDM Modeling Good Research Practices Task Force report on model parameter estimation and uncertainty (29). The cost-effectiveness acceptability curves (CEAC) was created to represent the probability that atezolizumab would be considered cost-effective at various willingness-to-pay (WTP) threshold levels.

## Results

### Base-Case Analysis

From the Chinese health sector perspective, in comparison with platinum-based chemotherapy, atezolizumab provided an additional 2.13 LYs, 1.27 LYs and 0.95 LYs in the high PD-L1 expression, high or intermediate PD-L1 expression, and any PD-L1 expression populations, respectively. Compared to chemotherapy, the incremental costs and QALYs of atezolizumab were $112,744.35 and 0.91 QALYs, $81,831.03 and 0.57 QALYs, and $70,346.51 and 0.42 QALYs for the high PD-L1 expression, high or intermediate PD-L1 expression, and any PD-L1 expression populations, respectively. Resulted in the ICERs for atezolizumab versus chemotherapy were $123,778.60/QALY in high PD-L1 expression populations, $142,827.19/QALY in high or intermediate PD-L1 expression populations, and $168,902.66/QALY in any PD-L1 expression populations. When the PAP was available, the marginal costs of atezolizumab were $47,742.13, $29,981.65, and $25,484.97, resulted in the ICERs for atezolizumab versus chemotherapy were $52,414.63/QALY, $52,329.73/QALY, and $61,189.66/QALY in the high PD-L1 expression, high or intermediate PD-L1 expression, and any PD-L1 expression populations, respectively (**Table 2**).

**Table 2:** Base-case results.

Strategies and Scenarios	Total cost, $	LYs	QALYs	ICER ($/QALY)
Without PAP				
High PD-L1 expression				
Chemotherapy	38,283.56	1.90	0.88	–
Atezolizumab	151,027.91	4.02	1.80	123,778.60
High or intermediate PD-L1 expression				
Chemotherapy	42,144.23	1.97	0.89	–
Atezolizumab	123,975.26	3.24	1.47	142,827.19
Any PD-L1 expression				
Chemotherapy	38,914.30	1.99	0.90	–
Atezolizumab	109,260.82	2.94	1.32	168,902.66
With PAP				
High PD-L1 expression				
Chemotherapy	38,283.56	1.90	0.88	–
Atezolizumab	86,025.68	4.02	1.80	52,414.63
High or intermediate PD-L1 expression				
Chemotherapy	42,144.23	1.97	0.89	–
Atezolizumab	72,125.89	3.24	1.47	52,329.73
Any PD-L1 expression				
Chemotherapy	38,914.30	1.99	0.90	–
Atezolizumab	64,399.27	2.94	1.32	61189.66

PAP, patient assistance program; LYs, life-years; QALYs, quality-adjusted life-years; ICER, incremental cost-effectiveness ratio; PD-L1, programmed death ligand 1.

### Sensitivity Analyses

**Figure 3:**
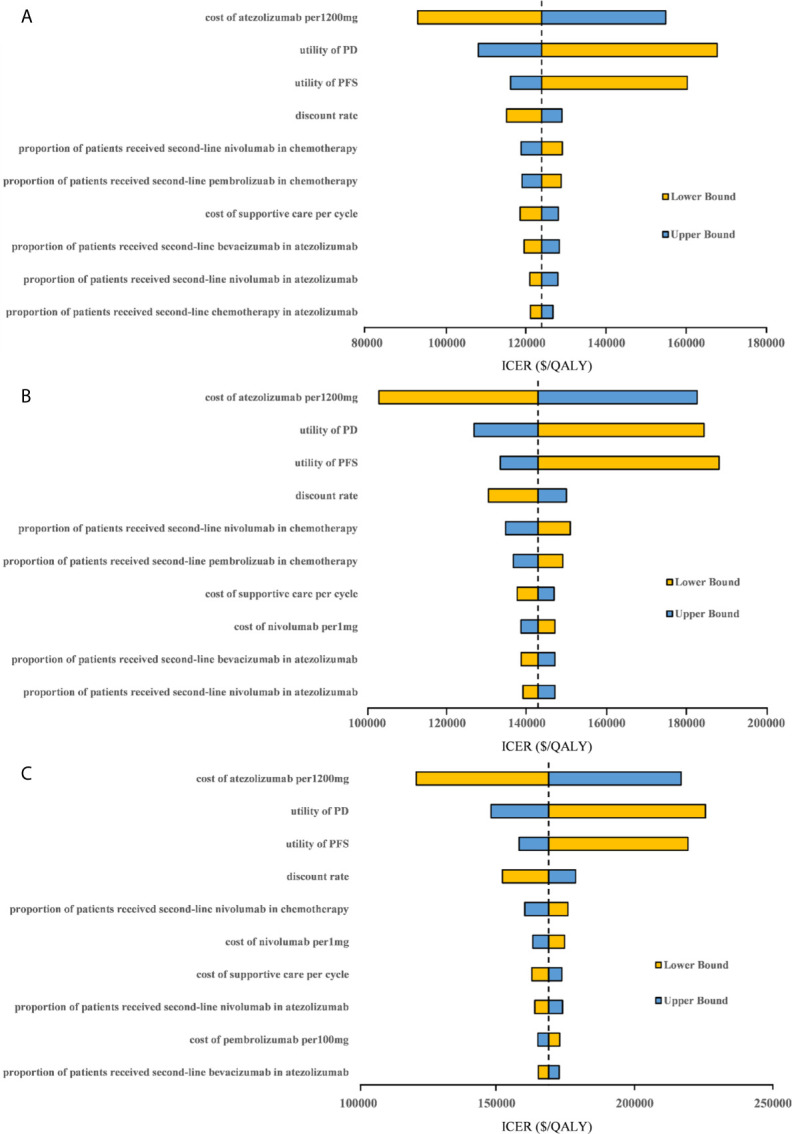
Tornado diagram of one-way sensitivity analyses with greatest influence parameters when the PAP was not available in **(A)** high PD-L1 expression populations, **(B)** high or intermediate PD-L1 expression populations and **(C)** any PD-L1 expression populations. PD-L1, programmed death ligand 1; PFS, progression-free survival; PD, progressed disease; ICER, incremental cost-effectiveness ratio.

**Figure 4:**
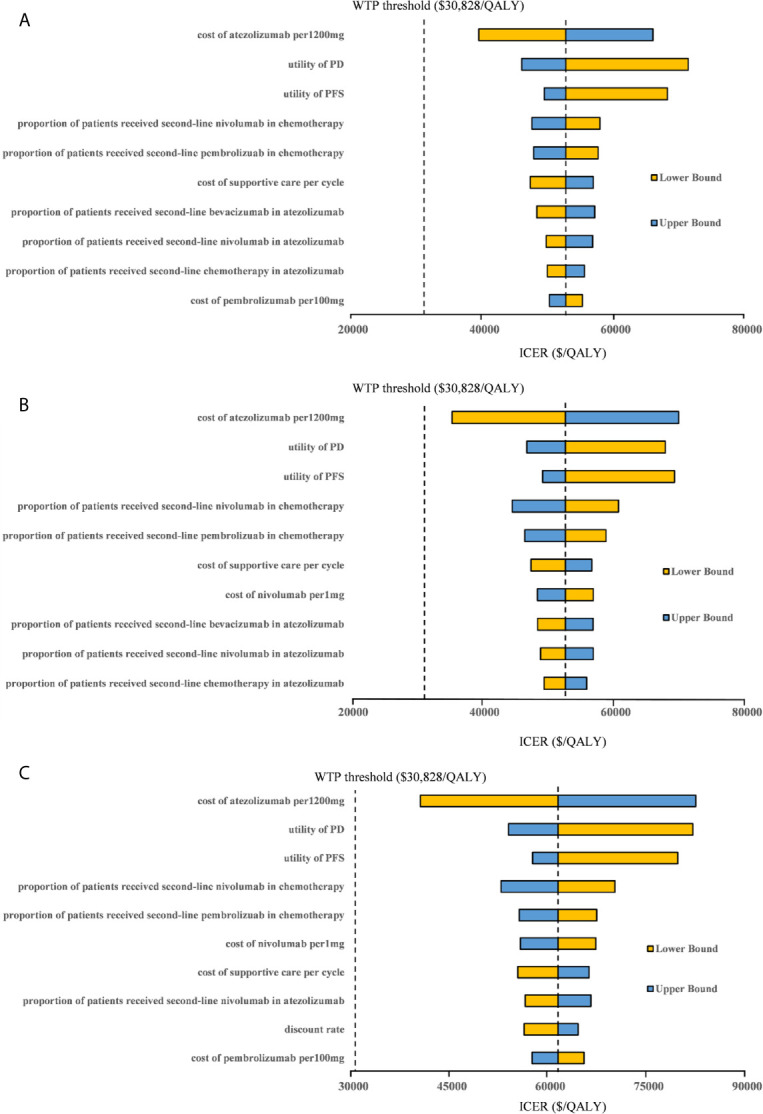
Tornado diagram of one-way sensitivity analyses with greatest influence parameters when the PAP was available in **(A)** high PD-L1 expression populations, **(B)** high or intermediate PD-L1 expression populations and **(C)** any PD-L1 expression populations. PD-L1, programmed death ligand 1; PFS, progression-free survival; PD, progressed disease; WTP, willingness-to-pay; ICER, incremental cost-effectiveness ratio.

One-way sensitivity analyses revealed that the parameters found to have the substantial influence on the ICERs were similar in the two scenarios regardless of the PD-L1 expression status: the cost of atezolizumab per 1200mg, the utility of PD, and the utility of PFS. Other parameters had a medium or small impact on the model outcome. Whether the PAP was available or not, none of the parameters leads to an ICER lower than the WTP threshold of $30,828 per additional QALY gained in the three categories of PD-L1 expression status populations (**Figures 3**, **4**).

**Figure 5:**
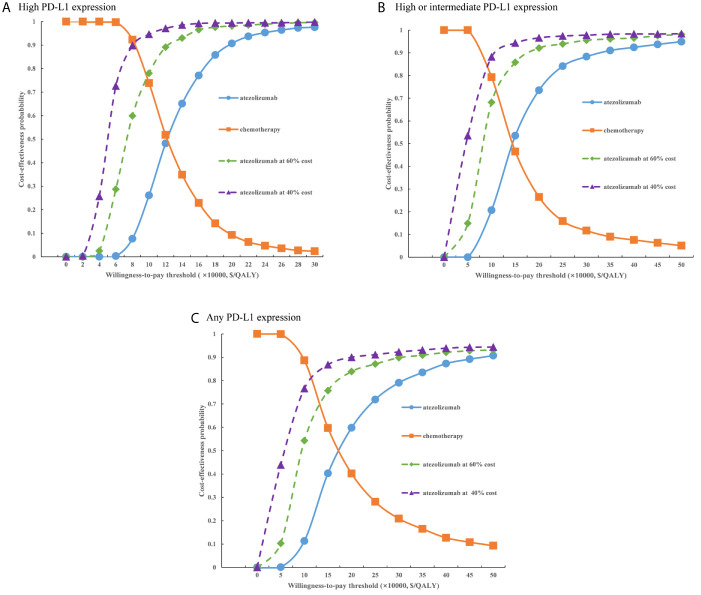
Cost-effectiveness acceptability curves of atezolizumab versus chemotherapy when the PAP was not available for **(A)** high PD-L1 expression populations, **(B)** high or intermediate PD-L1 expression populations and **(C)** any PD-L1 expression populations. PD-L1, programmed death ligand 1.

**Figure 6:**
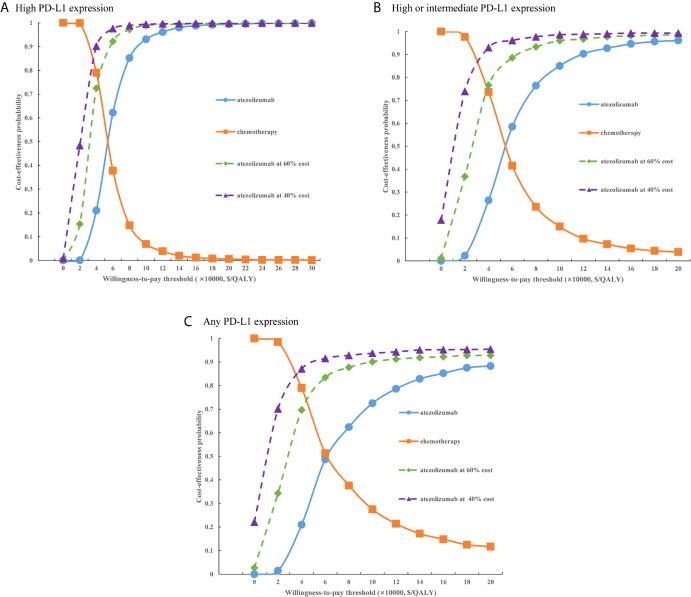
Cost-effectiveness acceptability curves of atezolizumab versus chemotherapy when the PAP was available for **(A)** high PD-L1 expression populations, **(B)** high or intermediate PD-L1 expression populations and **(C)** any PD-L1 expression populations. PD-L1, programmed death ligand 1.

For the probabilistic sensitivity analyses, when the atezolizumab PAP was not available, regardless of PD-L1 expression status, the cost-effectiveness acceptability curves (**Figure 5**) suggested that the probability of atezolizumab being cost-effective compared with chemotherapy was 0% at a WTP threshold of $30,828 per QALY gained in China, atezolizumab had 50% probability of being cost-effectiveness at an approximate WTP threshold of $126,000/QALY, $145,000/QALY, and $167,000/QALY of high PD-L1 expression, high or intermediate PD-L1 expression, and any PD-L1 expression populations, respectively. When the PAP was available, although the probabilities of atezolizumab being cost-effective were increase, they were not reached 15% yet in the three categories of PD-L1 expression status populations (**Figure 6**), and the WTP threshold of atezolizumab had 50% probability of being cost-effective was reduced to $53,000/QALY, $52,000/QALY, and $62,000/QALY of high PD-L1 expression, high or intermediate PD-L1 expression, and any PD-L1 expression populations, respectively.

## Discussion

Its motivated great interest for both patients and oncologists after the report of a clinical benefit from atezolizumab in the IMpower110 trial. However, the healthcare cost is dramatically increased with the widespread use of immunotherapy due to the high drug price, so it is necessary to evaluate its value in terms of both cost and efficacy especially for resource-limited countries such as China, and ensure to be sustainable for both reimbursement platform and healthcare system. To the best of our knowledge, this is the first study to evaluate the cost-effectiveness of atezolizumab versus chemotherapy as first-line treatment for metastatic non-small-cell lung cancer with different PD-L1 expression status from the Chinese health sector perspective. Our analysis demonstrated that, for the three categories of PD-L1 expression status populations, whether the PAP was available or not, atezolizumab as first-line treatment for metastatic NSCLC was unlikely to be cost-effective due to the unfavorable ICER when the WTP threshold was $30,828/QALY regardless of PD-L1 expression status, and the results were robust as shown by one-way sensitivity and probabilistic sensitivity analyses.

Currently, there is no relevant economic evaluation of atezolizumab versus chemotherapy as first-line treatment for patients with metastatic NSCLC. One recent analysis assessed the cost-effectiveness of pembrolizumab (30), a monoclonal antibody that against programmed cell death protein (PD-1), versus chemotherapy for previously untreated locally advanced or metastatic NSCLC with different PD-L1 tumor proportion scores from the perspective of Chinese payers based on the KEYNOTE-042 trial (12). Reported that pembrolizumab yielded additional costs and QALYs, compared with chemotherapy, were $65,322 and 1.79 QALYs, $51,196 and 1.21 QALYs, and $44,133 and 1.12 QALYs for three PD-L1 TPS populations (≥50%, ≥20% and ≥1%), resulting in an ICER of $36,493/QALY, $42,311/QALY, and $39,404/QALY, respectively. Which suggested that pembrolizumab was not cost-effective at a WTP threshold of $26,508/QALY in China, regardless of TPS. The results of one-way sensitivity analyses were consistent with our finding, the cost of immunotherapy agent, the utility of PD, and the utility of PFS were the most influential factors on the model outcomes. Notably, the ICERs were much lower than ours, that might be caused by the potential reasons: first, above study were performed from the different perspective to ours, this might make the measurement of the costs slightly different; second, the health state utility values were different, 0.761 for PFS and 0.687 for PD in the previously study, and 0.804 for PFS and 0.321 for PD in our model; third, although the drug acquisition cost were similar among atezolizumab and pembrolizumab in each cycle, pembrolizumab up to a maximum of 35 cycles, this could reduce the expenditure of the cost. Although immunotherapy drugs were different in IMpower110 and KEYNOTE-042 trial, atezolizumab and pembrolizumab demonstrated similar pricing and clinical benefits, so we considered their conclusion was inherent and comparable with our finding. Another similar analysis evaluated the cost-effectiveness of adding atezolizumab to first-line chemotherapy for advanced non-squamous NSCLC from the perspective of Chinese healthcare system based on the IMpower130 trial (31). The analysis revealed that atezolizumab plus chemotherapy was unlikely to be cost-effect due to the unfavorable ICER in comparison with chemotherapy in China, and the cost of atezolizumab had a substantial impact on the model results, which were consistent with our study. These similar experiences remind us that atezolizumab could not to be considered cost-effective for NSCLC in China whether alone or in combination with chemotherapy due to its high price.

At present, Chinese government adopted the way of national medical insurance negotiation with pharmaceutical companies by pharmacoeconomic evidence. The latest results of 2020 national medical insurance negotiation revealed that average price reduction drugs with successful negotiation was 50.64%, so we explored the effect of price reducing on the model results. When the PAP was not available, when the price of atezolizumab were decreased by 40%, 60%, the probability of atezolizumab being cost-effective was less than 30%, and atezolizumab would likely to be the cost-effective option for three categories of populations when the price was reduced approximately 75%. When the PAP was available, at the same price reduction ratio, the probability of atezolizumab being cost-effective would exceed 50% for the three categories of populations. These findings have reference value for guiding the rational allocation of the health resources by decision makers.

There are several limitations must be discussed in our study. First, the long-term clinical benefits beyond the observational time of the IMpower110 trial were extrapolated by fitting the parametric functions, this approach was an inevitable limitation of the study, which may cause bias between the model results and the real situation. Second, because of the absence of head-to-head trial, we did not evaluate other potential first-line competing treatments for metastatic NSCLC, such as pembrolizumab. Third, some key clinical cost, such as cost of supportive care per cycle, was gathered from the previously study rather than the real-world data (32–37), sensitivity analyses were performed to minimize the potential uncertainty of the model results. Finally, the costs for the management of grade 1/2 adverse events were excluded from the analysis, which might underestimate the economic results of atezolizumab, although sensitivity analyses revealed that only small influence of the model outcomes. Despite these limitations, we confident that the study accurately reflected the clinical conditions of metastatic NSCLC in China.

## Conclusion

In conclusion, our findings suggest that atezolizumab is unlikely to be a cost-effective option as first-line treatment for Chinese patients with metastatic NSCLC regardless of PD-L1 expression status. Reduce drug price and provide PAP for NSCLC patients can increase its cost-effectiveness.

## Data Availability Statement

The original contributions presented in the study are included in the article/**Supplementary Material**. Further inquiries can be directed to the corresponding author.

## Ethics Statement

This cost-effectiveness analysis was based on a literature review and modeling techniques, the study did not require approval from an Institutional Research Ethics Board.

## Author Contributions

GL and SK were involved in the design of the study. GL, SK, XW and FS were collected the data and performed the economic analysis. GL, SK, XW and FS drafted and critically revised the manuscript. All authors contributed to the article and approved the submitted version.

## Conflict of Interest

The authors declare that the research was conducted in the absence of any commercial or financial relationships that could be construed as a potential conflict of interest.
