# Supplementary material for: Cost-Effectiveness Analysis of Atezolizumab Versus Chemotherapy as First-Line Treatment for Metastatic Non-Small-Cell Lung Cancer With Different PD-L1 Expression Status
Source: Front Oncol. 2021 Apr 27;11:669195. doi: 10.3389/fonc.2021.669195 (PMC8111076; doi:10.3389/fonc.2021.669195)
Supplement: Supplementary file 1 [file Table_1.docx]

Supplementary Tables

**Supplementary Table 1 |** Summary of statistical goodness-of-fit of KM curves in IMpower110 trial.

**Supplementary Table 2 |** Model parameters: base-line values, ranges, and distributions for sensitivity analyses.

**Supplementary Table 1** **|** Summary of statistical goodness-of-fit of KM curves in IMpower110 trial.

|  | Exponential | Gamma | Weibull | Log-normal | Log-logistic | Gompertz |
| --- | --- | --- | --- | --- | --- | --- |
| Atezolizumab PFS curve of high PD-L1 expression patients | | | | | | |
| AIC | 485.563 | 486.167 | 484.843 | 475.719* | 477.889 | 477.812 |
| Atezolizumab OS curve of high PD-L1 expression patients | | | | | | |
| AIC | 395.917 | 394.356 | 393.969 | 392.768* | 393.375 | 393.937 |
| Chemotherapy PFS curve of high PD-L1 expression patients | | | | | | |
| AIC | 482.7 | 479.761 | 481.329 | 478.532 | 477.332* | 484.365 |
| Chemotherapy OS curve of high PD-L1 expression patients | | | | | | |
| AIC | 436.459 | 437.18 | 437.529 | 438.425 | 435.602* | 438.157 |
| Atezolizumab PFS curve of high or intermediate PD-L1 expression patients | | | | | | |
| AIC | 764.235 | 766.214 | 765.632 | 749.849* | 753.889 | 758.773 |
| Atezolizumab OS curve of high or intermediate PD-L1 expression patients | | | | | | |
| AIC | 614.286 | 614.635 | 614.55 | 617.618 | 614.607 | 614.21* |
| Chemotherapy PFS curve of high or intermediate PD-L1 expression patients | | | | | | |
| AIC | 775.563 | 761.328 | 764.923 | 761.358 | 757.88* | 774.531 |
| Chemotherapy OS curve of high or intermediate PD-L1 expression patients | | | | | | |
| AIC | 673.898 | 670.154 | 670.844 | 673.886 | 669.61* | 674.169 |
| Atezolizumab PFS curve of any PD-L1 expression patients | | | | | | |
| AIC | 1,279.027 | 1,280.768 | 1,279.148 | 1,252.157* | 1,257.725 | 1,266.549 |
| Atezolizumab OS curve of any PD-L1 expression patients | | | | | | |
| AIC | 1,016.553 | 1,018.328 | 1,018.139 | 1,017.887 | 1,016.26* | 1,016.703 |
| Chemotherapy PFS curve of any PD-L1 expression patients | | | | | | |
| AIC | 1,297.15 | 1,268.835 | 1,274.59 | 1,271.491 | 1,263.64* | 1,292.205 |
| Chemotherapy OS curve of any PD-L1 expression patients | | | | | | |
| AIC | 1,059.2 | 1,052.798 | 1,054.45 | 1,052.626 | 1,049.53* | 1,060.03 |

**, adopted parametric survival function in the model; AIC, Akaike information criterion; PFS, progression-free survival; OS, overall survival; PD-L1, programmed death ligand 1.*

**Supplementary Table 2** **|** Model parameters: base-line values, ranges, and distributions for sensitivity analyses.

| Parameters | Base-line value | Range | | Distribution | Reference |
| --- | --- | --- | --- | --- | --- |
|  |  | Minimum | Maximum |  |  |
| Cost inputs (US $) | | | | | |
| Atezolizumab per 1200mg | 4,754.31 | 3,565.73 | 5,942.89 | Gamma | Local charge |
| Carboplatin per 100mg | 10 | 7.5 | 12.5 | Gamma | Local charge |
| Cisplatin per 100mg | 26.53 | 19.90 | 33.16 | Gamma | Local charge |
| Pemetrexed per 500mg | 373.9 | 280.43 | 467.38 | Gamma | Local charge |
| Gemcitabine per 100mg | 50.2 | 37.65 | 62.75 | Gamma | Local charge |
| Docetaxel per 1mg | 3 | 2.25 | 3.75 | Gamma | Local charge |
| Pembrolizumab per 100mg | 2,597 | 1,947.75 | 3,246.25 | Gamma | Local charge |
| Nivolumab per 1mg | 15 | 11.25 | 18.75 | Gamma | Local charge |
| Bevacizumab per 100mg | 219.5 | 164.63 | 274.38 | Gamma | Local charge |
| Routine follow-up per cycle | 85.71 | 64.28 | 107.14 | Gamma | (32) |
| Supportive care per cycle | 338 | 159 | 476 | Gamma | (33) |
| Terminal care | 2,464.5 | 1,848.38 | 3,080.63 | Gamma | (34) |
| Anemia per event | 537 | 478 | 585 | Gamma | (35) |
| Neutropenia per event | 466 | 415 | 508 | Gamma | (35) |
| Thrombocytopenia per event | 6,397 | 5,117 | 7,676 | Gamma | (36) |
| Decreased platelet count per cycle | 6,397 | 5,117 | 7,676 | Gamma | (36) |
| Decreased neutrophil count per event | 466 | 0 | 1384 | Gamma | (37) |
| Febrile neutropenia per event | 953 | 715 | 1191 | Gamma | (37) |
| Utility inputs | | | | | |
| Utility of PFS | 0.804 | 0.536 | 0.883 | Beta | (27,28) |
| Utility of PD | 0.321 | 0.05 | 0.473 | Beta | (27,28) |
| Disutility of toxicities | | | | | |
| Anemia | -0.073 | -0.037 | -0.11 | Beta | (27) |
| Thrombocytopenia | -0.19 | -0.143 | -0.238 | Beta | (28) |
| Neutropenia | -0.2 | -0.15 | -0.25 | Beta | (28) |
| Decreased platelet count | -0.19 | -0.143 | -0.238 | Beta | (28) |
| Decreased neutrophil count | -0.2 | -0.15 | -0.25 | Beta | (28) |
| Febrile neutropenia | -0.42 | -0.315 | -0.525 | Beta | (28) |
| Risk of serious adverse events in atezolizumab group | | | | | |
| Anemia | 0.017 | 0.002 | 0.032 | Beta | (18) |
| Thrombocytopenia | 0.003 | 0 | 0.009 | Beta | (18) |
| Neutropenia | 0.007 | 0 | 0.017 | Beta | (18) |
| Risk of serious adverse events in chemotherapy group | | | | | |
| Anemia | 0.183 | 0.136 | 0.23 | Beta | (18) |
| Thrombocytopenia | 0.072 | 0.04 | 0.103 | Beta | (18) |
| Neutropenia | 0.175 | 0.129 | 0.22 | Beta | (18) |
| Decreased platelet count | 0.042 | 0.018 | 0.066 | Beta | (18) |
| Decreased neutrophil count | 0.038 | 0.015 | 0.061 | Beta | (18) |
| Febrile neutropenia | 0.034 | 0.012 | 0.056 | Beta | (18) |
| Atezolizumab subsequent therapy proportion in high PD-L1 expression populations | | | | | |
| Chemotherapy | 0.215 | 0.137 | 0.293 | Beta | (18) |
| Nivolumab | 0.019 | 0 | 0.045 | Beta | (18) |
| Bevacizumab | 0.084 | 0.031 | 0.137 | Beta | (18) |
| Chemotherapy subsequent therapy proportion in high PD-L1 expression populations | | | | | |
| Docetaxel | 0.184 | 0.107 | 0.26 | Beta | (18) |
| Nivolumab | 0.112 | 0.05 | 0.174 | Beta | (18) |
| Pembrolizumab | 0.173 | 0.098 | 0.248 | Beta | (18) |
| Bevacizumab | 0.071 | 0.021 | 0.122 | Beta | (18) |
| Atezolizumab subsequent therapy proportion in high or intermediate PD-L1 expression populations | | | | | |
| Chemotherapy | 0.265 | 0.198 | 0.332 | Beta | (18) |
| Nivolumab | 0.018 | 0 | 0.038 | Beta | (18) |
| Pembrolizumab | 0.012 | 0 | 0.029 | Beta | (18) |
| Bevacizumab | 0.066 | 0.028 | 0.104 | Beta | (18) |
| Chemotherapy subsequent therapy proportion in high or intermediate PD-L1 expression populations | | | | | |
| Docetaxel | 0.228 | 0.163 | 0.293 | Beta | (18) |
| Nivolumab | 0.154 | 0.098 | 0.21 | Beta | (18) |
| Pembrolizumab | 0.142 | 0.088 | 0.196 | Beta | (18) |
| Bevacizumab | 0.056 | 0.021 | 0.091 | Beta | (18) |
| Atezolizumab subsequent therapy proportion in any PD-L1 expression populations | | | | | |
| Chemotherapy | 0.278 | 0.225 | 0.331 | Beta | (18) |
| Nivolumab | 0.025 | 0.007 | 0.043 | Beta | (18) |
| Bevacizumab | 0.051 | 0.025 | 0.077 | Beta | (18) |
| Chemotherapy subsequent therapy proportion in any PD-L1 expression populations | | | | | |
| Docetaxel | 0.123 | 0.084 | 0.162 | Beta | (18) |
| Nivolumab | 0.159 | 0.116 | 0.202 | Beta | (18) |
| Pembrolizumab | 0.112 | 0.138 | 0.075 | Beta | (18) |
| Bevacizumab | 0.043 | 0.019 | 0.067 | Beta | (18) |
| Others | | | | | |
| Proportion of nonsquamous NSCLC in high PD-L1 expression populations | 0.765 | 0.681 | 0.849 | Beta | (18) |
| Proportion of nonsquamous NSCLC in high or intermediate PD-L1 expression populations | 0.716 | 0.647 | 0.786 | Beta | (18) |
| Proportion of nonsquamous NSCLC in any PD-L1 expression populations | 0.697 | 0.643 | 0.751 | Beta | (18) |
| Discount rate | 0.05 | 0 | 0.08 | - | (22) |

*PFS, progression-free survival; PD, progressed disease; PD-L1, programmed death ligand 1; NSCLC, non-small-cell lung cancer.*
